# Plant snRNP Biogenesis: A Perspective from the Nucleolus and Cajal Bodies

**DOI:** 10.3389/fpls.2017.02184

**Published:** 2018-01-04

**Authors:** Misato Ohtani

**Affiliations:** ^1^Graduate School of Biological Sciences, Nara Institute of Science and Technology, Ikoma, Japan; ^2^RIKEN Center for Sustainable Resource Science, Yokohama, Japan

**Keywords:** Cajal bodies, nucleolus, nucleus, pre-mRNA splicing, snRNA, snRNP

## Abstract

Small nuclear ribonucleoproteins (snRNPs) are protein–RNA complexes composed of specific snRNP-associated proteins along with small nuclear RNAs (snRNAs), which are non-coding RNA molecules abundant in the nucleus. snRNPs mainly function as core components of the spliceosome, the molecular machinery for pre-mRNA splicing. Thus, snRNP biogenesis is a critical issue for plants, essential for the determination of a cell’s activity through the regulation of gene expression. The complex process of snRNP biogenesis is initiated by transcription of the snRNA in the nucleus, continues in the cytoplasm, and terminates back in the nucleus. Critical steps of snRNP biogenesis, such as chemical modification of the snRNA and snRNP maturation, occur in the nucleolus and its related sub-nuclear structures, Cajal bodies. In this review, I discuss roles for the nucleolus and Cajal bodies in snRNP biogenesis, and a possible linkage between the regulation of snRNP biogenesis and plant development and environmental responses.

## Introduction

In eukaryotes, protein-coding genes contain non-coding sequence regions, called introns, as well as the coding regions, or exons. After transcription, the cellular machinery removes introns from primary transcripts and splices together the exons to generate the mature messenger RNA (mRNA). This process of pre-mRNA splicing is a critical step in mRNA metabolism and is carried out in nucleoplasmic regions by the spliceosome (reviewed by Will and Lührmann, 2011; Matera and Wang, 2014). Spliceosomes are large molecular machinery composed mainly of small nuclear ribonucleoproteins (snRNPs), which are protein–RNA complexes comprising small nuclear RNAs (snRNAs), a class of non-coding RNA molecules abundant in the nucleus, with specific snRNP-associated proteins. Major and minor forms of the spliceosome vary based on the type of snRNPs present and their target introns (reviewed by Patel and Bellini, 2008; Will and Lührmann, 2011; Matera and Wang, 2014; Lanfranco et al., 2017). The major spliceosome contains U1, U2, U4, U5, and U6 snRNPs as core components, while the minor spliceosome contains U11, U12, U4atac, U5, and U6atac snRNPs (Will and Lührmann, 2011; Matera and Wang, 2014). These UsnRNPs recognize intron sequences and cleave and join pre-mRNA by esterification reactions, resulting in the release of introns and splicing of exons into a complete mRNA (Will and Lührmann, 2011; Matera and Wang, 2014).

**FIGURE 1:**
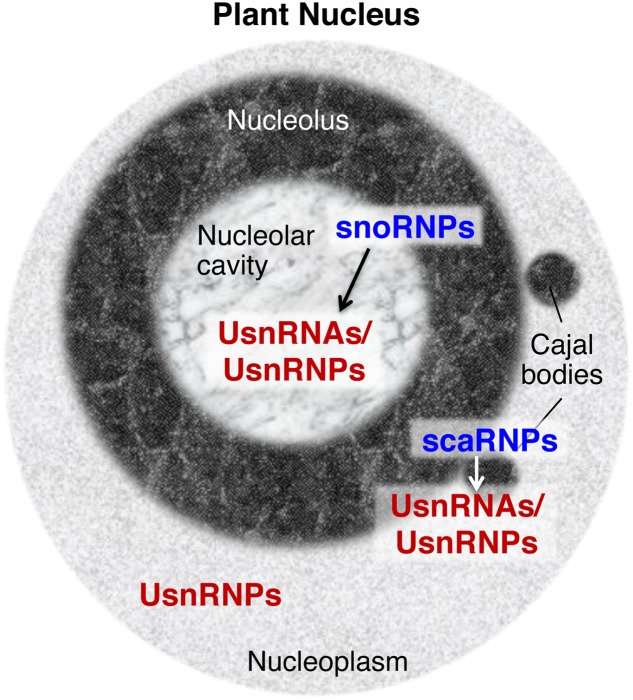
Small nuclear ribonucleoproteins (snRNPs) found in the plant nucleus. snRNPs distributed in specific nuclear domains have specific functions, such as the guidance of chemical modifications of UsnRNAs/UsnRNPs by snoRNPs or scaRNPs (indicated by arrows between snoRNPs and UsnRNAs/UsnRNPs, and scaRNPs and UsnRNAs/UsnRNPs), and pre-mRNA splicing by mature UsnRNPs in nucleoplasmic regions. UsnRNAs, uridylate-rich small nuclear RNAs, UsnRNPs, uridylate-rich small nuclear ribonucleoproteins, snoRNPs, small nucleolar ribonucleoproteins, scaRNPs, small Cajal body-specific ribonucleoproteins.

Other types of snRNPs regulate different aspects of RNA metabolism, such as the modification and processing of pre-ribosomal RNA (rRNA), and the modification of spliceosomal snRNAs (reviewed by Maxwell and Fournier, 1995; Kiss, 2004). The snRNPs for these RNA metabolic processes contain small nucleolar RNAs (snoRNAs) or small Cajal body-specific RNAs (scaRNAs) (**Figure 1**). These specific snRNAs contain conserved motifs, including box C, box D, box H, and box ACA; thus, snRNPs containing these snRNAs are also called snoRNPs or scaRNPs (Maxwell and Fournier, 1995; Jády et al., 2003; Kiss, 2004). The modifications and processing steps that are mediated by snoRNPs occur in the nucleolus and the steps mediated by scaRNPs occur in Cajal bodies, sub-nuclear structures that are physically and functionally associated with the nucleolus (**Figure 1**; reviewed by Bassett, 2012; Shaw and Brown, 2012; Love et al., 2017). snoRNPs or scaRNPs mediate the biogenesis of functional ribosomes and spliceosomal snRNPs. In addition, several species-specific snRNPs are known to have specific molecular functions: U7 snRNP functions in the 3′ processing of histone mRNA in metazoan cells (reviewed by Dominski and Marzluff, 2007) and 7SK snRNP is a critical regulator of the homeostasis and activity of P-TEFb, a key regulator of RNA polymerase ll transcription, in vertebrates (reviewed by Quaresma et al., 2016).

As snRNPs play fundamental roles in the regulation of gene expression, snRNP biogenesis is a critical regulatory step in determining cellular activity. The accumulated data indicate that snRNP biogenesis is a complex process and that the nucleolus, and Cajal bodies specifically, are pivotal elements of snRNP biogenesis (Patel and Bellini, 2008; Fischer et al., 2011; Matera and Wang, 2014; Lanfranco et al., 2017). Roles for such distinct nuclear compartments and subnuclear domains in the regulation of gene expression, cellular signaling, and stress responses have attracted attention over the years not only in animal cells (reviewed by Boulon et al., 2010), but also in plant cells (reviewed by Shaw and Brown, 2012; Ohtani, 2015; Love et al., 2017). However, it has been shown that the structures of nucleolus are different between animal and plant cells; for instance, plant nucleoli contain a specific structure known as the nucleolar cavity, which contains spliceosomal snRNAs and accumulates snoRNAs (**Figure 1**; Beven et al., 1995, 1996; reviewed by Shaw and Brown, 2012; Stepiński, 2014), suggesting that plant nucleoli could organize snRNP biogenesis in a plant-specific manner. Here, I provide an overview of current knowledge regarding spliceosomal snRNP biogenesis mechanisms, focusing on the nucleolus and Cajal bodies. I further discuss the linkage between snRNP biogenesis and plant development and environmental responses, from the viewpoint of nucleolus-based regulation of snRNP biogenesis.

## Current Model of Spliceosomal snRNP Assembly Based on Mammalian Studies

**FIGURE 2:**
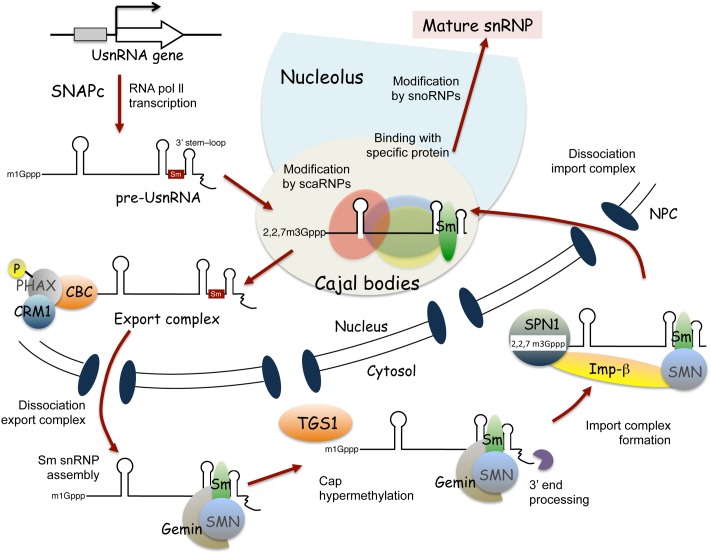
Current model of Sm class snRNP biogenesis in mammalian cells. Maturation of Sm class snRNPs involves nuclear and cytoplasmic steps. SNAPc, snRNA activating protein complex; CBC, cap-binding complex; PHAX, phosphorylated adapter RNA export; CRM1, chromosome region maintenance 1; TGS1, trimethylguanosine synthase 1; SPN, Snurportin; Imp-β, importin-β; NPC, nuclear pore complex.

Spliceosomal snRNP biogenesis has been extensively studied in mammalian cells (Patel and Bellini, 2008; Will and Lührmann, 2011; Matera and Wang, 2014; Lanfranco et al., 2017), and is known to vary for different snRNP species. For example, snRNPs containing RNA polymerase II-transcribed uridylate-rich small nuclear RNAs (UsnRNAs), such as U1, U2, U4, U5, U11, U12, and U4atac (also known as Sm class snRNAs based on sequence features and protein cofactors), undergo both nuclear and cytoplasmic maturation steps (**Figure 2**). By contrast, maturation of snRNPs containing RNA polymerase III-transcribed UsnRNAs, such as U6 and U6atac (also known as Sm-like class snRNAs), is completed within the nucleus (Patel and Bellini, 2008; Will and Lührmann, 2011; Matera and Wang, 2014; Lanfranco et al., 2017).

**Figure 2** shows a current model of Sm class snRNP assembly in mammalian cells. snRNP biogenesis is initiated by transcription of snRNAs through a complex called SNAPc (snRNA activating protein complex) (**Figure 2**; reviewed by Hernandez, 2001; Will and Lührmann, 2011; Matera and Wang, 2014; Ohtani, 2017). As for mRNAs transcribed by RNA polymerase II, the 5′ capping and 3′ cleavage of Sm class UsnRNAs occurs in a co-transcriptional manner. The transcriptional termination of Sm class UsnRNAs requires a large multiprotein complex, called the Integrator complex (reviewed by Chen and Wagner, 2010). The Integrator complex is thought to participate in the cleavage and polyadenylation of pre-snRNAs, acting with the 3′ box sequence, the Sm class UsnRNA-specific processing signal (Chen and Wagner, 2010; Matera and Wang, 2014). The 5′-linked *N*7-methyl guanosine (_m1_G) cap of transcribed pre-snRNA molecules is first recognized by the cap binding complex (CBC) (Izaurralde et al., 1994), followed by binding with the phosphorylated adaptor for RNA export (PHAX) (Ohno et al., 2000; Segref et al., 2001). In the nucleoplasm, the export receptor, chromosome region maintenance 1 (CRM1) (Fornerod et al., 1997), and the GTP-bound form of RAN GTPase interact with PHAX, to translocate the pre-snRNA molecules to the cytosol through the nuclear pore complex (Askjaer et al., 1999) (**Figure 2**). Interestingly, transcribed pre-snRNAs seem to pass through Cajal bodies before their nuclear export; pre-snRNA molecules with unprocessed 3′ extensions were found in Cajal bodies (Smith and Lawrence, 2000), and injection of pre-snRNA molecules into the nucleus showed the temporal localization of pre-snRNAs in Cajal bodies (Suzuki et al., 2010). Moreover, PHAX and CRM1 accumulate in Cajal bodies (Frey and Matera, 2001), suggesting that Cajal bodies also function in the formation of the export complex of pre-snRNA (Matera and Wang, 2014; Lanfranco et al., 2017).

In the cytoplasm, the export factors dissociate from the pre-snRNA molecule (Kitao et al., 2008). Next, the SMN-Gemins complex, which contains the SMN protein and seven Gemin proteins (Gemin 2–8), mediates the assembly of seven Sm core proteins at the Sm site of snRNA (Meister et al., 2001; Massenet et al., 2002; Narayanan et al., 2002; Pellizzoni et al., 2002; and the articles reviews in Lanfranco et al., 2017), to form Sm core snRNP, a stable ring-like structure (Raker et al., 1996; Kambach et al., 1999; Leung et al., 2011). The SMN–Gemins complex mediates the hypermethylation of the 5′ cap structure of snRNA by bridging it to trimethylguanosine synthase Tgs1 (Mouaikel et al., 2002, 2003), as well as the 3′ end trimming of snRNA (Matera and Wang, 2014; Lanfranco et al., 2017). The hypermethylated cap structure, known as the 2,2,7-trimethylguanosine cap structure, is recognized by the importer adaptor, Sniurpotin (SPN), that recruits the import receptor, Importin-β (Palacios et al., 1997; Huber et al., 1998). Sm core snRNPs are imported into the nucleus via importin-mediated transport through the nuclear pore complex. Following nuclear import, the SMN complex is immediately released from Sm core snRNPs (Matera and Wang, 2014; Lanfranco et al., 2017). Then, Sm core snRNPs accumulate in Cajal bodies, where additional modifications of the snRNAs and binding of snRNP-specific proteins occurs (Bassett, 2012; Matera and Wang, 2014; Lanfranco et al., 2017). *De novo* assembly and reassembly of U4-U6/U5 tri-snRNPs are thought to occur in Cajal bodies (Jády et al., 2003; Nesic et al., 2004; Schaffert et al., 2004; Matera and Wang, 2014). Finally, the matured spliceosomal snRNPs localize to nucleoplasmic speckle structures, called nuclear speckles, which facilitate pre-mRNA splicing (Will and Lührmann, 2011; Matera and Wang, 2014).

In the case of Sm-like class snRNP biogenesis, the pre-snRNA transcripts are localized in the nucleolus and then processed by 3′ trimming (Patel and Bellini, 2008). Further modification of the pre-snRNAs by snoRNPs and the binding of Lsm (like Sm) core proteins to the pre-snRNAs to yield stable ring-like structures also occur in the nucleolus (Achsel et al., 1999). U6 snRNAs transiently localize in the nucleolus after transcription, and then translocate into Cajal bodies (Lange and Gerbi, 2000), where U6 snRNPs are combined with U4 and U5 to form U4/U6.U5 tri-snRNPs (Schaffert et al., 2004). Thus, the maturation of Sm-like class snRNPs takes place in the nucleolus and Cajal bodies (Patel and Bellini, 2008; Matera and Wang, 2014).

In plants cells, snRNP biogenesis is thought to proceed via similar pathways as described in mammalian cells (Lorković and Barta, 2004; Shaw and Brown, 2012). However, experimental evidence pertaining to snRNP biogenesis processes in plant cells is limited, partly due to the absence of a suitable experimental system in which to examine the assembly and translocation of snRNAs and related proteins in plants, analogous to the *Xenopus* oocyte injection system (Cohen et al., 2009). In humans, spliceosome disorders have been linked to severe inherited diseases, such as spinal muscular atrophy, which is caused by reduced levels of SMN proteins (Matera and Wang, 2014; Lanfranco et al., 2017). Plant molecular genetics studies revealed that genes involved in snRNP biogenesis are important for plant development (Ohtani et al., 2008, 2010, 2013; Swaraz et al., 2011), circadian clock regulation (Deng et al., 2010; Hong et al., 2010; Sanchez et al., 2010; Schlaen et al., 2015), stress tolerance (Xiong et al., 2001; Zhang et al., 2011; Gao et al., 2017), and plant organ regeneration (Ohtani and Sugiyama, 2005; Ohtani et al., 2010, 2013) (reviewed by Staiger and Brown, 2013; Tsukaya et al., 2013; Shang et al., 2017; for the details, please see below), suggesting that snRNP biogenesis has indispensable roles in the differentiation and function of cells that are conserved between animals and plants.

## Roles for the Nucleolus and Cajal Bodies in Spliceosomal snRNP Biogenesis in Plant Cells

As described above, the nucleolus and Cajal bodies have pivotal functions in snRNP assembly in mammalian cells (**Figure 2**). Here, I provide an overview of what we know about the roles for the nucleolus and Cajal bodies in plant snRNP biogenesis.

### Chemical Modification of snRNAs Guided by snoRNAs and scaRNAs

Post-transcriptional modifications of snRNAs, guided by snoRNAs and scaRNAs, occur in the nucleolus and Cajal bodies (**Figure 1**; reviewed by Bassett, 2012; Love et al., 2017; Meier, 2017). These modifications are conserved among eukaryotes, including plants (Huang et al., 2005; Bassett, 2012), and are thought to convey the binding affinity of snRNPs for their substrate pre-mRNAs (Darzacq et al., 2002). After work in animal systems revealed scaRNAs, which carry a CB box that directs them to Cajal bodies, in addition to snoRNAs, which carry conserved box C, box D, box H, and box ACA (Jády and Kiss, 2001; Kiss et al., 2002), a genomic survey identified candidate scaRNAs in plant species (Marker et al., 2002). Experimental approaches also confirmed the localization of scaRNAs in Cajal bodies (Kim et al., 2010), suggesting that scaRNA-guided modification of snRNAs has important functions in plant cells (Bassett, 2012; Love et al., 2017).

In plant genomes, most snoRNA genes occur as polycistronic clusters (Leader et al., 1997; Brown et al., 2003; Chen et al., 2003). *In situ* hybridization analysis detected such polycistronic precursors of snoRNAs in the nucleolus and Cajal bodies (Shaw et al., 1998). Therefore, plant nucleoli function in the maturation of snoRNAs and in snoRNP assembly, to generate functional snoRNPs that modify snRNAs.

### Assembly of snRNA and snRNP-Specific Proteins

**FIGURE 3:**
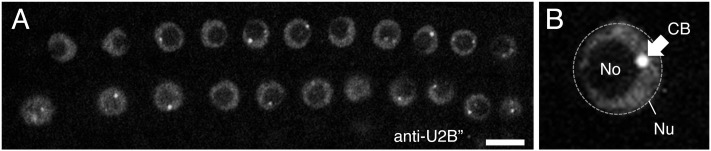
**(A)** Confocal image of Arabidopsis root nuclei with Cajal bodies, as visualized by immunostaining with anti-U2B” protein. **(B)** Shows a close-up view of the nucleus. Nu, nucleus (shown by a dotted line), No, nucleolus, and CB, Cajal body (shown by arrow). Scale bars, 10 μm.

Early studies in *Pisum sativum* (pea) showed that complexes harboring spliceosomal snRNPs were localized in the nucleolus and associated with sub-nuclear structures in close proximity to the nucleolus, later shown to be Cajal bodies (Beven et al., 1995, 1996). These observations have subsequently been supported by transient reporter assays in *Arabidopsis thaliana* (Arabidopsis) using fluorescent protein-tagged snRNP proteins, such as the U2 snRNP-specific protein U2B” (**Figure 3**; Boudonck et al., 1999; Collier et al., 2006) and U1 snRNP-specific proteins U1-70K, U1A, and U1C (Lorković and Barta, 2008). These snRNP-specific proteins have distinct distributions in nuclear regions; although all U2B”, U1-70K, U1A, and U1C proteins accumulate in both the nucleolus and Cajal bodies, but they co-localize only in Cajal bodies, U1-70K also localizes in nuclear speckles, the sites of pre-mRNA splicing, whereas the other U1 snRNP-specific proteins do not (Lorković and Barta, 2008). This observation suggests that the nuclear assembly pathway differs for different snRNP-specific proteins. Recently, Hyjek et al. (2015) described the dynamic assembly of U4 snRNPs during the first meiotic prophase in European larch microsporocytes. They showed that U4 snRNAs and Sm proteins have two distinct spatial distributions in the cytoplasm—diffuse or within distinct foci—which depend on the rate of *de novo* snRNP formation relative to the expression of U4 snRNAs and Sm proteins (Hyjek et al., 2015). Furthermore, they found that the distribution of snRNPs change dynamically in the nucleus; the size and number of Cajal bodies with U4 snRNP signals varied during meiotic prophase (Hyjek et al., 2015). Cajal body dynamics have been well established in Arabidopsis. Reporter analyses using the Cajal body marker proteins U2B” and/or Coillin (Boudonck et al., 1999; Collier et al., 2006) revealed that the number and size of Cajal bodies present varied depending on the cell cycle stage, cell type, and biotic and abiotic stresses present (**Figure 3A**; Shaw and Brown, 2012; Love et al., 2017), as shown in animal cells (Boulon et al., 2010). For example, the number of Cajal bodies was decreased in newly divided cells, and as the G1 phase progressed, the size of Cajal bodies increased (Boudonck et al., 1999). Cajal bodies were also disappeared immediately by heat shock treatment (Boudonck et al., 1999). In addition, Cajal bodies are reorganized into Cajal bodies-like structures by the infection of groundnut rosette virus (Kim et al., 2007a,b). These observations would partly reflect changes in *de novo* snRNP biogenesis activity under different cellular conditions.

The central role of the nucleolus in snRNP assembly was additionally supported by proteomics analysis of Arabidopsis nucleoli. Pendle et al. (2005) identified 217 proteins as nucleolar proteins containing proteins with ribosome-related functions, including ribosomal proteins, rDNA transcription regulators, and ribosome biogenesis-related proteins. In addition to these expected proteins, snRNP proteins and other spliceosomal proteins were identified (Pendle et al., 2005). A recent detailed proteome analysis of the nucleus and nucleolus of Arabidopsis identified 86 proteins annotated as pre-mRNA splicing-related factors, with snRNP proteins among them, and demonstrated that 49 of these were localized in both the nucleus and nucleolus (Palm et al., 2016). Some of such pre-mRNA splicing-related factors, e.g., RSZp22 (Tillemans et al., 2006) and eIF4A-III (Koroleva et al., 2009), have been shown to shuttle between the nucleus and nucleolus in response to cellular stresses. Thus, the pre-mRNA splicing-related factors detected in both the nucleus and nucleolus could be also under the regulation of active trafficking between the nucleus and nucleolus in plant cells. It was also suggested that post-translational modifications of nuclear proteins, including acetylation and phosphorylation, differ based on the localization of each protein. This implies that an important role of trafficking nuclear domains could be the regulation of post-translational modifications, and thus the regulation of protein activities, as shown for the phosphorylation-depending mobility of RSZp22 between nuclear domains (Tillemans et al., 2006). These proteome data also identified many “unknown proteins” and/or “plant-specific nucleolar proteins,” some of which were annotated as RNA-binding proteins (Pendle et al., 2005; Palm et al., 2016). It is possible that an unknown plant-specific regulatory system may contribute to snRNP assembly in the nucleolus. Arabidopsis ROOT INITIATION DEFECTIVE 1 (RID1), a nucleolus-localized DEAH-box RNA helicase, is a candidate to be one such factor. Although RID1 itself did not seem to be a direct part the spliceosome, pre-mRNA splicing was significantly affected in the *rid1-1* mutants (Ohtani et al., 2013). Future studies should examine the plant-specific aspects of snRNP assembly.

### Linkage between the Regulation of snRNP Biogenesis and Development, Growth, and Stress Responses in Plants

Since pre-mRNA splicing is critical for gene expression, severe disorders of snRNP biogenesis are expected to be lethal for eukaryotic cells. Indeed, knock-out mutations of snRNA biosynthesis genes and essential components of snRNPs result in gametophyte or embryo lethality in Arabidopsis (Tsukaya et al., 2013). However, molecular genetic studies have revealed that snRNP biogenesis-related genes could be related to specific physiological processes in Arabidopsis (Tsukaya et al., 2013; Ohtani, 2015), such as circadian clock regulation, abiotic and biotic stress responses, and plant regeneration (Staiger and Brown, 2013; Tsukaya et al., 2013; Ohtani, 2015; Shang et al., 2017). For example, Arabidopsis homologs of PROTEIN ARGININE METHYLTRANSFERASE 5 (PRMT5) and GEMIN2, the genes essential for the formation of the SMN-Gemins complex, which is required for the binding of Sm core proteins to snRNAs, are important for circadian clock regulation (Deng et al., 2010; Hong et al., 2010; Sanchez et al., 2010; Schlaen et al., 2015). Mutations of LSM4 and LSM5, which encode essential core proteins of Sm-like class snRNPs (**Figure 2**; Achsel et al., 1999), and of Tgs1, which hypermethylates the 5′ cap of snRNAs (**Figure 2**; Mouaikel et al., 2002), were reported to enhance plant sensitivities to abiotic stresses, such as salt, drought, and cold stress (Xiong et al., 2001; Zhang et al., 2011; Gao et al., 2017). Moreover, SHOOT REDIFFERENTIATION DEFECTIVE 2 (SRD2), a subunit of the snRNA-specific transcription activator complex, SNAPc (**Figure 2**), is required for *in vitro* dedifferentiation and organogenesis (Ohtani and Sugiyama, 2005; Ohtani et al., 2015; Ohtani, 2015, 2017). The disorders of the corresponding mutants can be explained by the misregulation—due to altered RNA processing—of specific genes involved in key processes, i.e., circadian rhythms, stress responses, and auxin polar transport (Xiong et al., 2001; Deng et al., 2010; Hong et al., 2010; Ohtani et al., 2010; Sanchez et al., 2010; Zhang et al., 2011; Schlaen et al., 2015).

Recent studies using pre-mRNA splicing inhibitors targeting a subunit of U2 snRNPs indicated that the global inhibition of pre-mRNA splicing primarily triggered transcriptomic changes resembling those in abiotic stress responses. These changes were partly mediated by a disturbance in the signaling pathway of abscisic acid, a key phytohormone in the stress response. The inhibitor was expected to affect all splicing events equally; however, the effects of inhibitor treatment were shown to differ for different genes (AlShareef et al., 2017; Ling et al., 2017). Thus, the impact of pre-mRNA splicing inhibition has to be interpreted from the wider perspective of mRNA turnover, considering factors such as transcriptional kinetics and/or mRNA stability. Notably, poly(A)-mRNA molecules accumulate in Cajal bodies long after their synthesis in plants (Niedojadło et al., 2014), implying that Cajal bodies function in the (pre-)mRNA metabolism, possibly serving as a storage site and/or quality control checkpoint. Notably, the plant nucleolus contains the proteins involved in nonsense-mediated decay/mRNA surveillance (Pendle et al., 2005), as well as aberrantly spliced mRNAs (Kim et al., 2009). Thus, it can be speculated that Cajal bodies might fine-tune the rate of snRNP biogenesis in response to mRNA usage and/or might function in quality control during the translation. Detailed analyses of such possibilities are required, to further obtain clues as to why specific molecular pathways place high demands on *de novo* snRNP biogenesis.

## Conclusion and Perspectives

The functions of snRNPs, such as in pre-mRNA splicing, are essential for gene expression in eukaryotic cells. The biogenesis of snRNPs is a highly complicated process, including both nuclear and cytoplasmic steps for snRNA modification and protein–snRNA interaction (**Figures 1**, **2**). Molecular genetic work has indicated that eukaryotic cells place high demands on *de novo* snRNP biogenesis for specific molecular pathways, such as the differentiation of motor neurons in human (Lanfranco et al., 2017), and environmental responses in plants (Shang et al., 2017), suggesting that each step of snRNP biogenesis could function as a kind of molecular node between cellular activity and cellular circumstance. Advanced visualization studies have suggested that there are dynamic changes in distributions of snRNP-related factors, depending on the rate of *de novo* snRNP biogenesis, in plant cells (Lorković and Barta, 2008; Hyjek et al., 2015). Thus, the spatiotemporal regulation of snRNP biogenesis could be a critical aspect of a cell’s response to its needs.

As discussed in this review, the nucleolus plays central roles not only in ribosome biosynthesis, but also in snRNP biogenesis. Current work has expanded our knowledge of the functions of the nucleolus. Now we know that the nucleolus is not a static structure, but an active and dynamic functional structure (Smoliński et al., 2007; Boulon et al., 2010; Shaw and Brown, 2012; Love et al., 2017). In addition to ribosome biosynthesis and snRNPs biogenesis, the nucleolus is involved in mRNA surveillance, i.e., mRNA quality control by the nonsense-mediated decay system (Pendle et al., 2005; Kim et al., 2009) and in microRNA biogenesis (Pontes and Pikaard, 2008). Thus, the nucleolus is filled with all sorts of functional RNAs and their interacting proteins. Within the nucleolus, these RNAs and proteins must be well assembled to properly function, according to cellular conditions. In line with this idea, we can consider the nucleolus as a center of RNA processing that links cellular conditions with cellular activity (Shaw and Brown, 2012).

From the perspective of the evolution of eukaryotic cellular partitioning, the establishment of the nucleolus as a center of RNA processing represents a major innovation that prevents the diffusion of RNP macromolecules, leading to the enhancement of RNA processing efficiency (Collins et al., 2009). We know that many features of the nucleolus are conserved between animals and plants. However, plant-specific features of nucleolus also exist; for example, the abundant accumulation of mRNAs within the nucleolus might be a plant-specific phenomenon (Shaw and Brown, 2012). The structure of the nucleolus also greatly differs between animals and plants (Shaw and Brown, 2012; Stepiński, 2014). Thus, after the separation of the animal and plant lineages, the functionally specialized compartmentation within the nucleolus might have progressed differently between these lineages.

The view that the plant nucleolus is a center of RNA processing raises the question as to how plant nucleoli manage to organize multiple, complicated processes, including ribosome biosynthesis, snRNP biogenesis, mRNA surveillance, and microRNA biogenesis. One solution could be the partitioning of specific RNA processing activity via sub-nuclear structures. If these sub-nuclear structures are flexibly formed and demolished, as shown in Cajal bodies, plant nucleoli can accelerate specific activity of RNA processing when necessary (Kanno et al., 2016). Future work on RNA processing mechanisms within the nucleolus will answer this important question.

## Author Contributions

MO designed this review work, assembled and analyzed the related papers, performed the experiments, and wrote the manuscript.

## Conflict of Interest Statement

The author declares that the research was conducted in the absence of any commercial or financial relationships that could be construed as a potential conflict of interest.
